# Virtual Reality for Pain Relief in the Emergency Room (VIPER) – a prospective, interventional feasibility study

**DOI:** 10.1186/s12873-022-00671-z

**Published:** 2022-06-21

**Authors:** T. Birrenbach, F. Bühlmann, A. K. Exadaktylos, W. E. Hautz, M. Müller, T. C. Sauter

**Affiliations:** Department of Emergency Medicine, Inselspital, University Hospital Bern, Freiburgstrasse 16C, 3010 Bern, Switzerland

**Keywords:** Pain management, Anxiety, Virtual reality simulation, Emergency department, e-Health, Telemedicine, Telehealth

## Abstract

**Background:**

Pain is one of the most common, yet challenging problems leading to emergency department (ED) presentation, despite the availability of a wide range of pharmacological therapies. Virtual reality (VR) simulations are well studied in a wide variety of clinical settings, including acute and chronic pain management, as well as anxiety disorders. However, studies in the busy environment of an adult ED are scarce.

The aim of this study is to explore the feasibility and effectiveness of a VR simulation for pain and anxiety control in a convenience sample of adult ED patients presenting with traumatic and non-traumatic pain triaged 2–5 (i.e., urgent to non-urgent) with a pain rating of ≥ 3 on a numeric rating scale (NRS 0–10).

**Methods:**

Prospective within-subject, repeated measures interventional feasibility pilot study at a Swiss University ED. The intervention consisted of a virtual reality simulation in addition to usual care. Pain and anxiety levels were measured using a verbally administered numeric rating scale (NRS) before and after the intervention. Information on patient experience was collected using established rating scales.

**Results:**

Fifty-two patients were enrolled. The most common pain localisations were extremities (*n* = 15, 28.8%) and abdomen (*n* = 12, 23.1%). About one third of patients presented with trauma-associated pain (*n* = 16, 30.8%). Duration of pain was mainly acute (< 24 h) (*n* = 16, 30.8%) or subacute (> 24 h) (*n* = 32, 61.5%). The majority of patients were triage category 3, i.e. semi-urgent (*n* = 48, 92.3%). Significant reduction in pain (NRS median pre-VR simulation 4.5 (IQR 3–7) vs. median post-VR simulation 3 (IQR 2–5), *p* < 0.001), and anxiety levels (NRS median pre-VR simulation 4 (IQR 2–5) vs. median post-VR simulation 2 (IQR 0–3), *p* < 0.001) was achieved, yielding moderate to large effect sizes (Cohen’s d estimate for pain reduction = 0.59 (95% CI 0.19—0.98), for anxiety level on NRS = 0.75 (95% CI 0.34—1.15). With medium immersion and good tolerability of the VR simulation, user satisfaction was high.

**Conclusions:**

Virtual reality analgesia for pain and anxiety reduction in the busy setting of an ED is feasible, effective, with high user satisfaction. Further randomized controlled studies are needed to better characterize its impact on pain perception and resource utilization.

**Supplementary Information:**

The online version contains supplementary material available at 10.1186/s12873-022-00671-z.

## Background

Pain is one of the most common problems leading to emergency department (ED) presentation. However, pain management in the ED remains challenging [1–3]. Many barriers to effective pain management in the ED have been reported, and include the individual pain level of the patient (e.g., the highly subjective experience of pain) as an important influencing factor [1, 4]. A wide range of pharmacological therapies including both opioid and non-opioid medications exist and are broadly applied in the ED via different routes of application, yet there are specific side effects and contraindications to consider [3]. Furthermore, EDs are a major source of opioid prescription and thus, in some countries, contribute to the rising opioid dependency crisis [5, 6]. Non-pharmacological therapies (e.g. relaxation techniques, traditional distraction, and transcutaneous electrical nerve stimulation are recommended as well, but are oftentimes underused in the busy acute-care setting of an ED [3].

Virtual Reality (VR) affects the visual and the auditory senses allowing immersion in a virtual world thanks to a VR headset, giving participants the illusion of “being there” in the 3 dimensional computer generated world as if it is a place they are visiting. The mechanism of how VR works to alleviate pain is only partially known. The gate control theory postulates pain perception to be modulated by interaction among different neurons. VR or other stimuli might lead to a closure of the neural gateways, thus reducing pain perception [7]. In addition to distraction [8], there are novel mechanisms for VR treatment in pain, such as producing neurophysiologic changes related to conditioning and exposure therapies [9], or regulating autonomic, affective (mood, anxiety), and evaluative (subjective pain and enjoyment rating) responses associated with acute pain [10]. fMRI studies have demonstrated that VR can reduce pain comparable to a moderate dose of opioids [11, 12]. The effects of VR simulations are well studied in a wide variety of clinical settings, including acute and postoperative [13–21], as well as chronic pain management [13, 17, 22], neuropathic pain [23], in patients undergoing invasive procedures [22, 24, 25], and for treatment of burn patients [26, 27]. Furthermore, there is a growing body of evidence for the application of VR simulation especially in the pediatric population (burn pain, painful procedures, chemotherapy, anxiety, and palliative care [11, 21, 28–32]. Additionally, VR simulation can be used for the treatment of anxiety disorders [18, 20, 30, 33].

Studies in the busy environment of an adult ED are scarce [34], and mainly focus on procedural analgesia during painful medical interventions.

Thus, we conducted a within-subject, repeated measure interventional feasibility pilot study to investigate:

i) The feasibility of deployment of a VR simulation in the busy setting of the ED for an adult population presenting with traumatic or non-traumatic pain ≥ 3 on a numerical rating scale (NRS) (0–10).
ii) The effectiveness of the VR simulation in pain and anxiety control. Impact of gender and pain location on response.
iii) The acceptance of the VR simulation in the study population and patient experience (user satisfaction, simulator sickness, sense of presence and immersion).

## Methods

### Study design, setting, and ethical approval

This is a prospective self-controlled interventional feasibility pilot study at the ED of the University Hospital of Bern, Switzerland. Our ED is a tertiary care centre, caring for a patient population of around 2 million and treating over 45,000 adult patients each year with an interdisciplinary team [35, 36].

The study took place from March 22^nd^, 2021 until July 9^th^, 2021, during daytime hours depending on availability of the study investigator.

All patients admitted to the ED were triaged by registered nurses using the Swiss triage scale, a five-level triage scale with high inter-rater and intra-rater reliability [37]. Chief complaints, objective parameters (vital signs), and key questions are used to stratify the risk: 1—life-threatening emergencies requiring immediate care, 2—urgent conditions requiring medical evaluation within 20 min, 3 – semi-urgent conditions, requiring medical evaluation within 2 h, 4 – non-urgent conditions and 5—follow-ups.

This study was classified as a quality evaluation study by the local institutional review board (Kantonale Ethikkommission Bern (KEK), BASEC-Number Req-2020–01,266).

### Inclusion/exclusion

We recruited a convenience sample (*n* = 52) of adult (≥ 18 years of age) ED patients triaged 2–5 on the Swiss triage scale, i.e. excluding critically ill/injured patients in shock, with a pain rating of NRS ≥ 3 on a numeric rating scale (0–10) who presented to the ED with the following complaints: traumatic and non-traumatic musculosceletal pain (back, pelvis, neck, extremities), abdominal or chest pain, or headache.

#### Exclusion criteria were as follows:

- Hemodynamically unstable patient (e.g., planned for admission to the intensive care unit or deemed unstable by the physician in charge)
- Patients without decision-making capacity or with communication deficits (e.g., hearing loss, patient unable to communicate in German at a level sufficient to give informed consent and answer questions about pain and anxiety)
- Altered mental status (e.g., intoxication, cognitive impairment, acute confusional state, acute psychosis, acute stroke, and developmentally delayed patients).
- History of drug abuse
- Patient unable to use VR due to vision problems (e.g., blindness or without his/her glasses).
- Patient suffering from epilepsy or other sensitivity to flashing light or motion
- Pregnancy or other medical condition prone to severe nausea and vomiting
- Patient suffering from claustrophobia
- Patients on non-invasive ventilation and patients requiring oxygen delivered by face mask
- Patients requiring droplet, aerosol and contact precautions
- Patient with injuries/skin affections (rashes, open wounds) to face/neck including traumatic brain injury that prevents the use of the VR headset
- Imprisoned patients
- Patient who participated in this study at a previous consultation
- Refusal to participate in the study

### Baseline data

Sociodemographic data (gender, age, highest level of education, need to wear glasses, prior experience with VR), clinical data regarding pain presentation (localisation, association with trauma or tumor, presence of neuralgic pain, duration (acute < 24 h, subacute > 24 h, chronic > 3 months) and triage level according to the Swiss triage scale) [37] were collected in a survey.

### Intervention

The study investigator (FB) informed the patient about the study aims, handed out the information form and ensured the absence of contraindications, responded to the patient's questions and collected the patient’s free, informed and expressed consent.

The intervention consisted of the application of the Healthymind VR simulation (HEALTHY MIND, Paris, France), using a Pico G2 4 K VR headset (Pico Interactive Inc., San Francisco, California, USA) with resolution of 1920 × 2160 and a diagonal field of view of 101 degrees and Bose Quiet Comfort 35 II noise-cancelling headphones (Bose Corporation, Framingham, Massachusetts, USA) as an adjunct to usual care in the ED. Pain medication was administered throughout the patient’s stay in the ED as it would be during a usual ED visit. In our ED, analgesia is administered according to a clearly defined standard and protocol, which was also adhered to unchanged during the study. If necessary the VR simulation was interrupted, so that medication (or other medical interventions) could be provided. The content of the simulation has been developed by a private company (HEALTHY MIND, Paris, France) and is a registered Class I medical device that is commercially available. The company was not involved in any aspects of the study. The immersive, but not interactive experience, consists of a contemplative relaxing landscape accompanied by a sound universe specifically composed to relax the patient. The patient could choose between either a forest or beach setting (Supplemental materials Figs. 1 and 2). The duration of the VR simulation was aimed at 20 min. If necessary, the simulation could be interrupted for important medical procedures or because of patient preference.

The application was controlled by the study investigator using an android tablet (Samsung Galaxy Tab A 2019, 4G; Samsung Electronics Co.,Ltd., Suwon, South Korea). The study investigator (FB) was always present during the simulation and worked with ED staff as necessary to ensure the patient was receiving appropriate clinical care throughout the duration of the intervention.

### Primary and secondary outcomes

#### Primary outcome measures

##### Pain reduction

Effectiveness of the VR simulation on the patients' self-assessment of their current pain intensity by a verbally administered numeric rating scale (NRS from 0 to 10 integers) immediately pre- and post-intervention. This scale has been demonstrated to be a valid and reliable tool for the assessment of acute pain in the ED [38].

#### Secondary outcome measures

##### Anxiety reduction

Effectiveness of VR simulation on the patients' self-assessment of their current anxiety measured on a verbally administered numeric rating scale (NRS from 0 to 10 integers) immediately pre- and post-intervention. Furthermore, the validated Patient-Reported Outcomes Measurement Information System (PROMIS®) Anxiety short form 8a (8 Items) was filled out by the patient immediately before and after the intervention [39, 40].

To evaluate the raw score of the anxiety intensity on the PROMIS Anxiety short form 8a, the value of the response options ranging from one to five for each of the eight items is summarized (raw score ranges from 8 to 40, with higher scores indicating higher anxiety levels).

##### Vital signs

Vital signs (blood pressure, heart rate, respiratory rate) were collected within a 10-min time-frame before and after the simulation.

#### Patient experience

##### Motion sickness

Motion sickness was assessed on a verbally administered numeric rating scale (NRS 0 to 10), immediately before and after the procedure.

“Visually-induced motion sickness” was assessed with four-items (nausea, headache, blurred vision, dizziness) according to the Simulator Sickness Questionnaire (SSQ) adapted from Kennedy et al. (total score ranges from 1 = no simulator sickness to 5 = strong simulator sickness) [41].

##### Sense of presence and immersion

Presence and immersion in the virtual world was determined according to the 6-item questionnaire developed by Slater-Usoh-Steed (total score ranges from 1 = no immersion to 7 = full immersion) [42].

##### User satisfaction

User satisfaction was assessed using a 7-item questionnaire (1: I liked the experience with the simulation; 2: The headset and headphones were comfortable; 3: The audio quality was pleasant; 4: The image quality was pleasant; 5: The simulation improved my discomfort; 6: I would use this simulation again with these complaints; 7: I would recommend this simulation to others. Answers on a Likert Scale from 1 = totally disagree to 5 = totally agree) directly after the procedure.

### Confounders

Information on the analgesic and anxiolytic medication administered in the ED was collected from the electronic patient healthcare dossier (e-care).

### Statistical analysis

Data was analysed in Stata® 16.1 (StataCorp, The College Station, Texas, USA).

Baseline characteristics are presented as numbers and percentage or median and interquartile range (IQR) using descriptive statistics as appropriate. Comparisons between two independent groups (e.g. male vs. female) were carried out by Chi-square or Wilcoxon rank sum test depending on variable (categorial or continuous).

Pre- and post-simulation comparisons (e.g. pain) were performed with McNemars test or Wilcoxon signed rank test. Incomplete variables are indicated. No data were imputed. Only complete data pairs could be evaluated. A *p*-value < 0.05 was considered significant. There was no p-value adjustment in this exploratory data analysis. Effect sizes with 95% confidence intervals (CI) for pain and anxiety levels before and after the simulation were determined by Cohen’s d. Effect size was determined as follows: Cohen’s d < 0.5 small, 0.5 – 0.8 moderate, and > 0.8 large.

## Results

### Recruitment, missing data

**Fig. 1:**
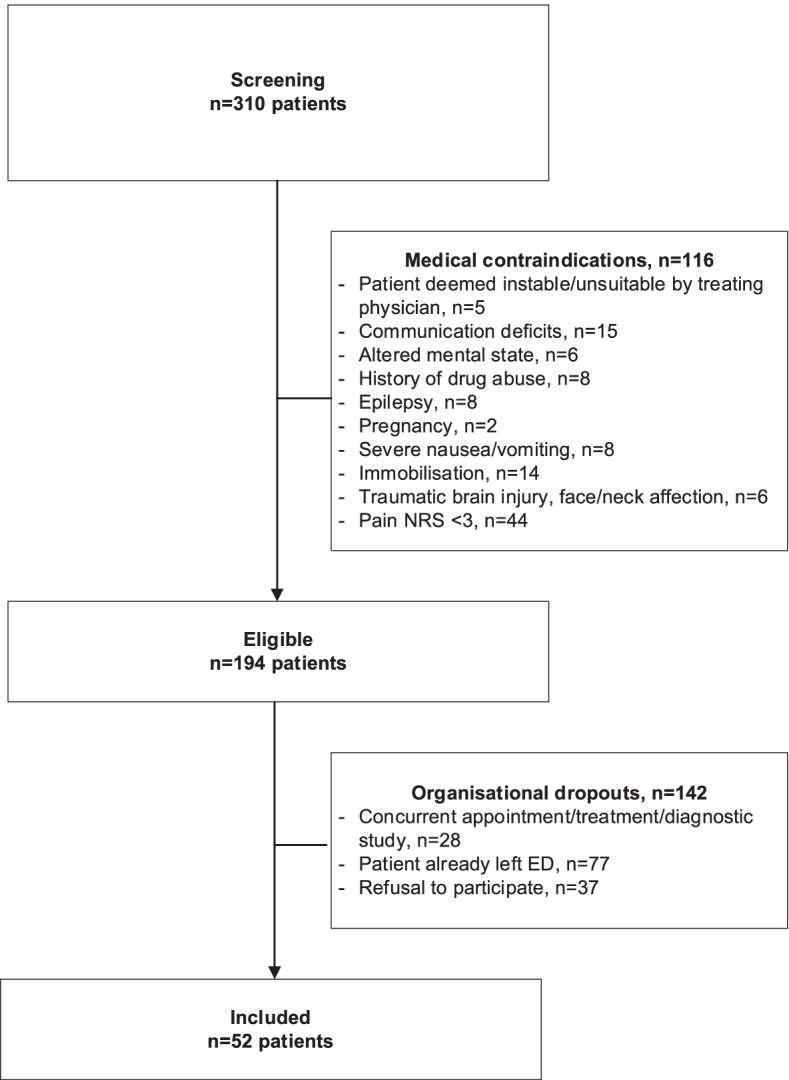
Flowchart

A total of 310 ED patients were screened, with in 194 patients meeting eligibility criteria (Flowchart Fig. 1). After determining eligibility, 28 patients were missed by the study investigator due to patient undergoing a clinical evaluation, diagnostic study or procedure, 77 patients had already left the ED, and 37 patients refused to participate in the study. Finally, 52 patients were enrolled in this study, and all but 2 patients included completed at least 10 min of the VR simulation (two patients required urgent medical intervention leading to discontinuation of the simulation after one and two minutes, respectively).

### Characteristics of patient population

Baseline characteristics of the patient population are detailed in Table 1. Overall, 52 patients were recruited, 32 females and 20 males. The most common pain localisations were extremities (*n* = 15, 28.8%), abdomen (*n* = 12, 23.1%), head (*n* = 8, 15.4%), back, and chest (each *n* = 5, 9.6%). About one third of patients presented with trauma-associated pain (*n* = 16, 30.8%). No patient presented with tumor-associated pain. Duration of pain was mainly acute (< 24 h) (*n* = 16, 30.8%) or subacute (> 24 h) (*n* = 32, 61.5%). Over ninety percent of patients were triaged level 3 (*n* = 48, 92.3%). Median pain level before VR on a NRS from 0 to 10 was 4.5 (IQR 3–7), median anxiety level was 4 (IQR 2–5). Median anxiety level on the 8-item PROMIS questionnaire was 14 out of a maximum of 40 points (IQR 11–21), with significant differences between females (median 18, IQR 12–26) and males (median 12.5, IQR 9.5–15), *p* = 0.002.

**Table 1:** Baseline characteristics of the patient population as a whole and according to gender

	**Total**		**Gender**				
	**Number**	**(*n* = 52)**	**Female**	**(*n* = 32)**	**Male**	**(*n* = 20)**	***P*-value**
**Sociodemographic characteristics**							
**Age, med (IQR)**	42	(35.5–55.5)	41.5	(36–56)	42.5	(34.5–56)	0.829
**Gender, n (%)**							
Male	20	(38.5)	0	(0.0)	20	(100.0)	
Female	32	(61.5)	32	(100.0)	0	(0.0)	
**Highest education level, n (%)**							
No formal education	1	(1.9)	1	(3.1)	0	(0.0)	
Obligatory	10	(19.2)	4	(12.5)	6	(30.0)	
Secondary	18	(34.6)	14	(43.8)	4	(20.0)	
Tertiary	23	(44.2)	13	(40.6)	10	(50.0)	0.184
**Glasses, n (%)**	22	(42.3)	15	(46.9)	7	(35.0)	0.399
**Prior experience in VR, n (%)**	22	(42.3)	13	(40.6)	9	(45.0)	0.756
**Pain characteristics**							
**Pain localisation, n (%)**							
Extremities	15	(28.8)	9	(28.1)	6	(30.0)	
Abdomen	12	(23.1)	5	(15.6)	7	(35.0)	
Head	8	(15.4)	7	(21.9)	1	(5.0)	
Back	5	(9.6)	3	(9.4)	2	(10.0)	
Chest	5	(9.6)	4	(12.5)	1	(5.0)	
Neck	2	(3.8)	2	(6.2)	0	(0.0)	
Pelvis	1	(1.9)	0	(0.0)	1	(5.0)	
Other	4	(7.7)	2	(6.2)	2	(10.0)	0.324
**Trauma-associated pain, n (%)**	16	(30.8)	11	(34.4)	5	(25.0)	0.476
**Tumor-associated pain, n (%)**	0	(0.0)	0	(0.0)	0	(0.0)	
**Neuralgiform pain, n (%)**	1	(1.9)	1	(3.1)	0	(0.0)	0.425
**Duration of pain, n (%)**							
Acute < 24 h	16	(30.8)	9	(28.1)	7	(35.0)	
Subacute > 24 h	32	(61.5)	21	(65.6)	11	(55.0)	
Chronic > 3 months	4	(7.7)	2	(6.2)	2	(10.0)	0.726
**Triage level, n (%)**							
2 (urgent)	2	(3.8)	2	(6.2)	0	(0.0)	
3 (semi-urgent)	48	(92.3)	29	(90.6)	19	(95.0)	
4 (non-urgent)	1	(1.9)	0	(0.0)	1	(5.0)	
5 (follow-up)	1	(1.9)	1	(3.1)	0	(0.0)	0.321
**Pain level, pre [NRS 0–10], med (IQR)**	4.5	(3–7)	5	(3–7.5)	4	(3–5.5)	0.255
**Pain bearable, pre, n (%)**	45	(86.5)	27	(84.4)	18	(90.0)	0.563
**Anxiety level, pre [NRS 0–10], med (IQR)**	4	(2–5)	5	(2–6)	3.5	(1–5)	0.205
**PROMIS anxiety total, pre, med (IQR), [scores ranging from 8–40]**^a^	14	(11–21)	18	(12–26)	12.5	(9.5–14)	0.002
**Pain level, pre [NRS 0–10], med (IQR)**	4.5	(3–7)	5	(3–7.5)	4	(3–5.5)	0.255

^a^Number *n* = 51, due to incomplete questionnaire

*Abbreviations*: *IQR* Interquartile range, *med* Median, *PROMIS* Patient-Reported Outcomes Measurement Information System

### Simulation details

Technical details of the VR simulation are described in Supplement Table 1. 28.8% of simulations were interrupted at least once, mainly due to medical interventions. Mean simulation time was 20 min. No significant differences regarding gender were found.

### Pain and anxiety reduction

Significant reduction in pain (NRS median pre-simulation 4.5 (IQR 3–7) vs. median post-simulation 3 (IQR 2–5), *p* < 0.001), and anxiety levels (NRS median pre-simulation 4 (IQR 2–5) vs. median post-simulation 2 (IQR 0–3), *p* < 0.001; PROMIS median pre-simulation 14 (IQR 11–21) vs. median post-simulation 8 (IQR 8–11), *p* < 0.001) was achieved (Table 2). Effect sizes were moderate to large (Cohen’s d estimate for pain reduction = 0.59 (95% CI 0.19—0.98), Cohen’s d estimate for anxiety level on NRS = 0.75 (95% CI 0.34—1.15), Cohen’s d estimate for anxiety level on PROMIS = 1.15 (95% CI 0.72—1.57).

**Table 2:** Pain and anxiety levels before and after the VR simulation. Median (IQR) if not mentioned otherwise

	**Pre**			**Post**			
	**N**	**Result**		**N**	**Result**		***P*-value**
**Pain level, [NRS 0–10]**	52	4.5	(3–7)	50	3	(2–5)	< 0.001
**Anxiety level, [NRS 0–10]**	52	4	(2–5)	49	2	(0–3)	< 0.001
**PROMIS anxiety total, scores ranging from 8–40**	51	14	(11–21)	49	8	(8–11)	< 0.001
**Pain bearable, n (%)**	52	45	(86.5)	48	46	(95.8)	0.125

*Abbreviations*: *IQR* Interquartile range, *med* Median, *PROMIS* Patient-Reported Outcomes Measurement Information System

No significant differences in vital parameters were observed pre- and post-simulation (Supplement Table 2).

**Fig. 2:**
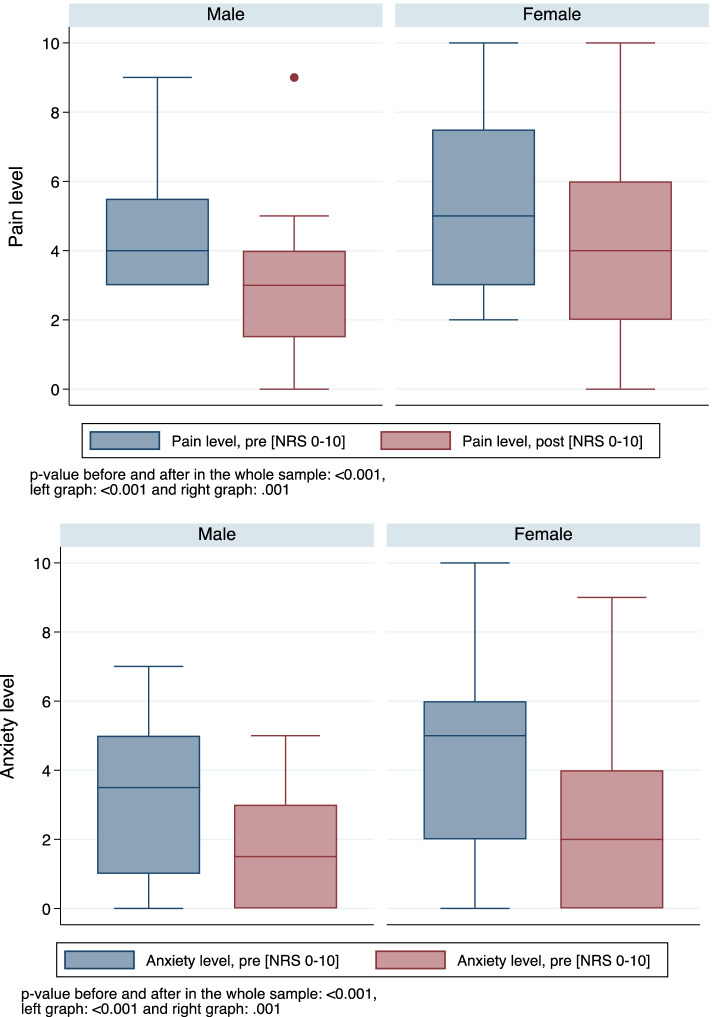
Pain and anxiety levels (NRS 0–10) before and after the VR simulation according to gender

Pain and anxiety levels according to gender are detailed in Fig. 2.

**Fig. 3:**
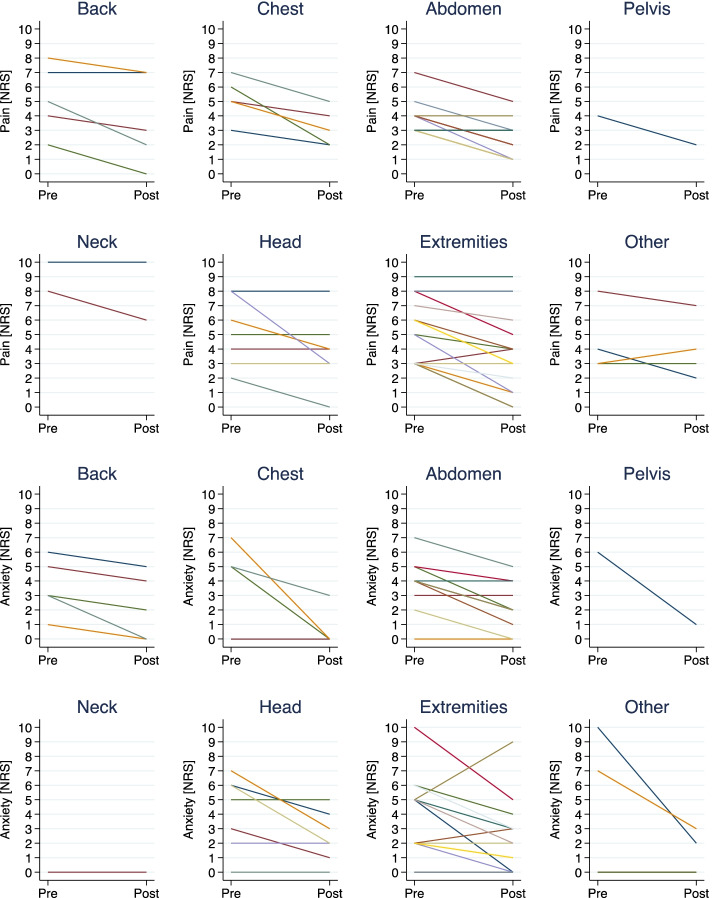
Pain and anxiety levels (NRS 0–10) before and after the VR simulation according to pain location

Effects of the VR simulation on pain and anxiety levels (NRS 0–10) according to pain location are detailed in Fig. 3. No significant differences of the effectiveness of the VR simulation on pain and anxiety levels according to gender were found (*p* = 0.324).

Forty-two percent of patients (*n* = 22) received analgesics before the simulation, and only 11% (*n* = 6) received opioids. Compared to males, the proportion of females receiving pain medications and opioids was significantly higher (*p* = 0.010 and *p* = 0.040, respectively) (Supplement Table 3). Only three patients received analgesics during the intervention.

**Fig. 4:**
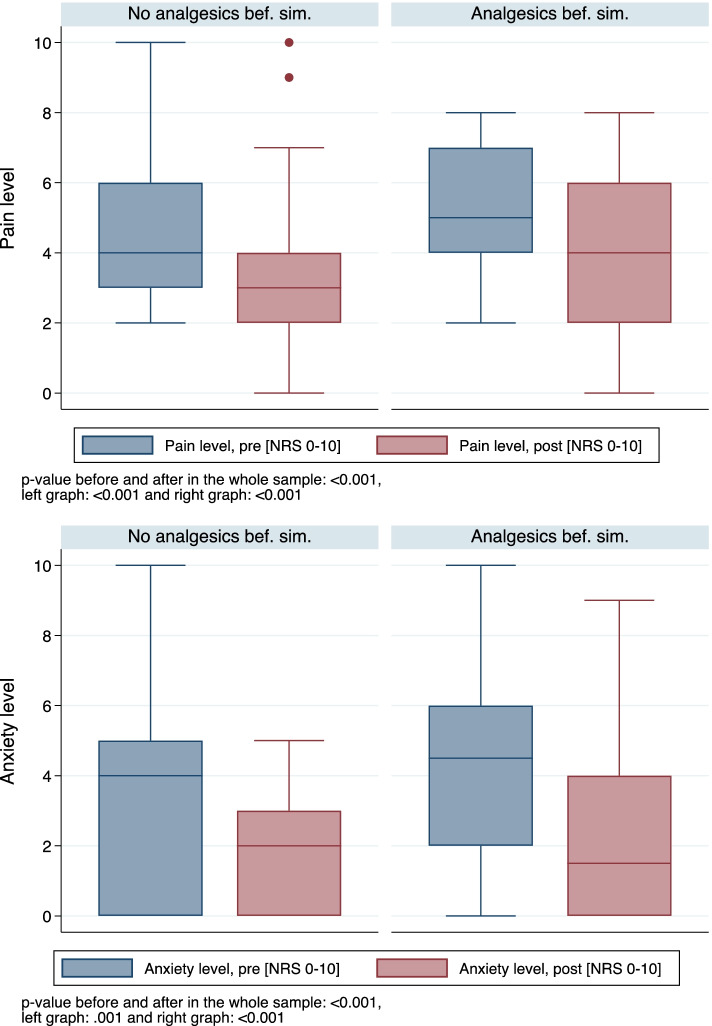
Pain and anxiety levels (NRS 0–10) before and after the VR simulation according to administration of analgesics before the simulation

A significant reduction of pain and anxiety levels was achieved using adjunctive VR (Fig. 4).

### Patient experience

**Fig. 5:**
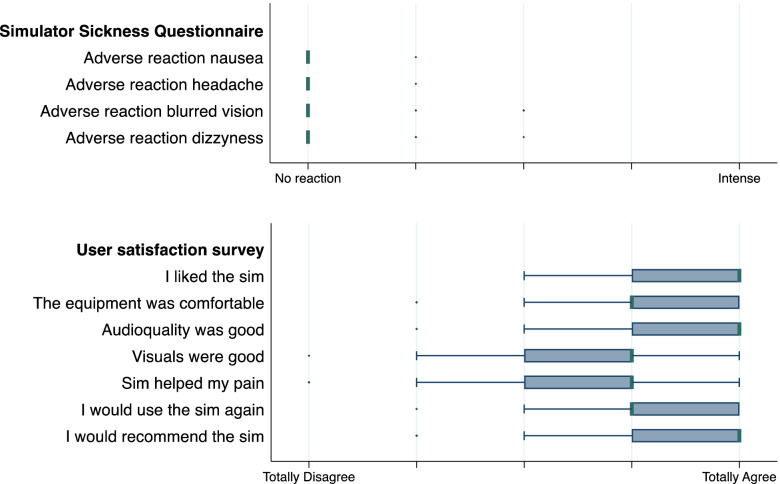
Results of the Simulator Sickness Questionnaire and User Satisfaction Survey. The medians of the distribution are indicated by a bold line. Outliers, i.e., the last observed value ≤ 1.5 × interquartile range above the upper quartile/below the lower quartile, are indicated with a point marker

Presence and immersion in the virtual world according to the questionnaire of Slater, Usoh and Steed was “medium illusion of being there” (median 3.8 on a scale from 1 to 7, IQR (2.8–4.7). The 4-item Simulator Sickness Questionnaire in the intervention group revealed a good tolerability of the VR simulation. Motion sickness measured on a NRS (0–10) was significantly lower after than before the simulation (median pre-simulation 0.5, IQR 0–3, median post-simulation 0, IQR 0–1, *p* < 0.001). The overall responses in the user satisfaction survey were positive. Over half of the patients agreed to the statement that the simulation helped with their pain (*n* = 26, 53.1%), and over 90% would recommend the simulation (*n* = 46, 93.9%) (Fig. 5). No significant differences in user satisfaction were found according to gender.

## Discussion

### Summary

In this prospective within-subject, repeated measure interventional pilot study, adjunctive virtual reality simulation proved to be feasible, effective and safe in a convenience sample of adult patients presenting with traumatic and non-traumatic pain, even in the busy setting of an adult ED, to reduce pain and anxiety.

Our patients mainly presented with acute and subacute musculosceletal, and abdominal pain, but also headache. We could demonstrate a significant pain and anxiety reduction after the 20 min VR distraction simulation, regardless of gender. Furthermore, the simulation proved effective regardless of the administration of analgesics before the intervention. We found good tolerability of the VR simulation with a high user satisfaction.

### Recruitment

Mosadegi et al., studied feasibility of an immersive VR experience in hospitalized patients. Recruitment in their study proved crucial with only a small number of patients ultimately able and willing to participate. Consistent with the “digital divide” for emerging technologies, they found younger patients were more willing to participate, a finding we did not replicate [43, 44]. In the current study, patient recruitment was much less of an issue. The more advanced VR system used in the current study may contribute to the higher patient acceptance. The main reasons for exclusion were organizational (i.e. concurrent appointment, diagnostic study, medical treatment, or the fact that the patient had already left the ED after consultation). As we did not want to disturb normal patient diagnostic and treatment flow in our busy ED with the study intervention, this finding was not unexpected.

### Effectiveness on pain and anxiety

The current study found a significant reduction in pain and anxiety levels through the VR simulation. Our results confirm the little existing evidence regarding effectiveness of VR on pain reduction in the ED. Sikka et al. were the first to study of the effect of VR in ED patients with acute pain [34]. In their convenience sample of 100 adult patients presenting to an urban academic ED with undifferentiated pain of at least 3 on a NRS (0–10), around 2/3 of the population were women and African American, presenting mainly with musculosceletal, abdominal and back pain. The mean VR application time in their study was shorter (6.9 ± 4.4 min). Both their reported pain and anxiety scores dropped significantly from pre- to post-intervention (pain 7.16 ± 2.5 to 6.49 ± 2.7, *p* < 0.0001; anxiety 2.06 ± 0.8 to 1.81 ± 0.8, *p* < 0.0001) with good tolerability. However, as a main limitation, they did not provide information regarding concurrent analgesic medication administered. In our study, we noticed a significant reduction of pain and anxiety levels, regardless of administration of analgesics before the simulation.

Spiegel et al. conducted a prospective randomized controlled study (VR on demand vs. specialized television program) in hospitalized patients with pain scores of at least 3 out of 10 on a NRS and achieved a similar level of pain reduction (mean within-subject difference in immediate pre- and post-intervention pain scores in the VR group (-1.72 points; SD 3.56), with a significantly greater pain reduction in the VR group than in the control group (-0.46 points; SD 3.01) [14]. Although the effect of VR on pre- and post-simulation pain scoring was statistically significant, the absolute reduction in pain scores in these and our patients was relatively small but clinically meaningful. The minimal clinical important difference (MCID) on the NRS usually lies between 1 and 2 points [45, 46]. A recent study demonstrated that patients perceived a change of 1.65 points on NRS in their pain severity as meaningful [47].

We did not find a significant change in physiological parameters, in line with the current lack of firm evidence that VR therapy can affect autonomic arousal or demonstrate its analgesic properties through modulation of these parameters [20].

Increased levels of anxiety can lead to worsening pain perception, decreased pain threshold and less cooperative patients [20, 48]. We found significantly higher levels of anxiety in women, and such gender differences have been reported elsewhere [49]. The ability of VR to remove patients from the anxiety-inducing clinical environment of a busy ED and immerse them in a relaxing virtual environment reduced both the associated pain and anxiety regardless of gender in our patients. One might also speculate, that VR pain and anxiety control can potentially help reduce the patients risk of developing chronic pain or post-traumatic stress symptoms in the future.

### Patient experience

Our simulation was well tolerated, with a low incidence of side effects such as nausea, headache, blurred vision or dizziness or motion sickness, in accordance with previous other reports [20, 21]. The use of calm VR worlds may have helped minimize simulator sickness in the current study.

Strong evidence has also shown that the quality of VR and the amount of immersion as well as interactivity delivered by the VR technology directly correlates with the measured quantity of analgesic effect [8, 9, 48]. Even though the sense of presence and immersion measured according to Slater in our patients was medium (probably due to interruptions, and the busy setting ED), our simulation still proved to be effective and safe with good subjective user satisfaction. In comparison to other VR systems, the interactivity of our simulation was deliberately low, in order not to overburden the VR inexperienced person. Further studies are needed to determine the optimal degree of presence and immersion as well as interactivity possible to achieve good results in the busy setting of an adult ED.

### Limitations

These results need to be interpreted with some reservations. First, this was a single tertiary care academic center study with a limited number of participants, that may be affected by selection bias, and thus have impact on generalizability. Furthermore, as this was a feasibility study, we included an uncontrolled convenience sample (no randomization or blinding), and results may been influenced by performance bias. We cannot exclude the novelty effect of using a previously unknown technique [50], but other studies have shown VR continued to reduce pain with repeated use [32]. This research was limited to patients with a self-reported pain level of 3 or greater on a NRS but did not control for baseline medication, final diagnosis, or pharmacological interventions. But note that we report pre- and post-intervention measures for each individual patient, and only three patients received additional pain medications during the VR.

Although premature to recommend VR as standard of care in the ED, the current results warrant consideration of a multicenter study in a larger patient group. Further comprehensive and structured research is needed to measure additional confounders and outcomes to identify optimal use of the VR simulation application in the ED setting. In addition, studies on the exact mechanism of action of the VR simulation beyond simple distraction would be valuable.

## Conclusion

VR simulation for pain and anxiety reduction in the busy setting of an ED is feasible, effective, and safe. Further and larger randomized controlled studies are needed to better characterize its impact on pain perception and resource utilization.

## Supplementary Information

**Additional file 1:** **Supplement table 1.** Technical details of the VR simulation. **Supplement table 2.** Vital parameters before and after the VR simulation. **Supplement table 3** Analgesics administered before, during or after the simulation, according to gender. **Supplement figure 1.** Beach simulation. **Supplement figure 2.** Forest simulation.

## Data Availability

Data contain potentially identifying or sensitive patient information. Data used in this study are available upon reasonable request from the corresponding author at the Emergency Department of the University Hospital Bern, Switzerland to researchers eligible under Swiss legislation to work with codified personal health care data. Eligibility will be determined by Cantonal ethics committee Bern.

## Abbreviations

CI: Confidence interval
ED: Emergency department
IQR: Interquartile range
MCID: Minimal clinical important difference
Med: Median
NRS: Numeric rating scale
PROMIS: Patient-Reported Outcomes Measurement Information System
SSQ: Simulator sickness questionnaire
VR: Virtual reality
