# Supplementary material for: Virtual Reality for Pain Relief in the Emergency Room (VIPER) – a prospective, interventional feasibility study
Source: BMC Emerg Med. 2022 Jun 21;22:113. doi: 10.1186/s12873-022-00671-z (PMC9210626; doi:10.1186/s12873-022-00671-z)

# Supplemental materials

### Supplement table 1: Technical details of the VR simulation

|  | **N** | **Result** |  |
| --- | --- | --- | --- |
| **Simulation duration [minutes], med (IQR)** | 52 | 20 | (20-20) |
| **Simulation module, n (%)** | 52 |  |  |
| Beach |  | 24 | (46.2) |
| Forrest |  | 28 | (53.8) |
| **Study location, n (%)** | 52 |  |  |
| Main ED |  | 25 | (48.1) |
| Minors area |  | 27 | (51.9) |
| **Interruptions** |  |  |  |
| Interruptions during simulation , n (%) | 52 |  |  |
| no |  | 37 | (71.2) |
| yes |  | 15 | (28.8) |
| Interruptions during simulation [number], med (IQR) | 52 | 0 | (0-1) |
| Interruption due to patient preference, n (%) | 52 |  |  |
| no |  | 48 | (92.3) |
| yes |  | 4 | (7.7) |
| Interruption due to medical intervention, n (%) | 52 |  |  |
| no |  | 43 | (82.7) |
| yes |  | 9 | (17.3) |
| Interruption due to technical problem, n (%) | 52 |  |  |
| no |  | 49 | (94.2) |
| yes |  | 3 | (5.8) |
| Interruption due to other reason, n (%) | 52 |  |  |
| no |  | 51 | (98.1) |
| yes |  | 1 | (1.9) |
| **Abortion** |  |  |  |
| Premature abortion of simulation, n (%) | 52 |  |  |
| no |  | 42 | (80.8) |
| yes |  | 10 | (19.2) |
| Abortion due to patient preference, n (%) | 52 |  |  |
| no |  | 46 | (88.5) |
| yes |  | 6 | (11.5) |
| Abortion due to medical intervention, n (%) | 52 |  |  |
| no |  | 48 | (92.3) |
| yes |  | 4 | (7.7) |
| Abortion due to technical problem, n (%) | 52 |  |  |
| no |  | 52 | (100.0) |
| Abortion due to other reason, n (%) | 52 |  |  |
| no |  | 51 | (98.1) |
| yes |  | 1 | (1.9) |

Abbreviations: IQR: interquartile range, med: median

### Supplement table 2: Vital parameters before and after the VR simulation

| **Vital parameters** | **Before simulation** | | | **After simulation** | | |  |
| --- | --- | --- | --- | --- | --- | --- | --- |
|  | **N** | **Result** |  | **N** | **Result** |  | **P-value** |
| **SBP**, [mmHg] , med (IQR) | 50 | 130 | (119-147) | 45 | 137 | (123-142) | .986 |
| **DBP**, [mmHg], med (IQR) | 50 | 78.5 | (70-83) | 45 | 77 | (70-84) | .688 |
| **Puls,** [per minute] , med (IQR) | 50 | 72 | (65-80) | 45 | 72 | (64-79) | .986 |
| **Respiratory rate**, [per minute] , med (IQR) | 49 | 14 | (12-16) | 47 | 14 | (12-16) |  |

Abbreviations: DBP: diastolic blood pressure, IQR: interquartile range, med: median, SBP: systolic blood pressure

### Supplement table 3: Analgesics administered before, during or after the simulation, according to gender

|  | **Total** |  |  | **Gender** |  |  |  |  |
| --- | --- | --- | --- | --- | --- | --- | --- | --- |
|  | **N=52** |  |  | **Female** | **(n=32)** | **Male** | **(n=20)** | **P-value** |
| **Analgesics before simulation, n (%)** | 52 |  |  |  |  |  |  |  |
| no |  | 30 | (57.7) | 14 | (43.8) | 16 | (80.0) |  |
| yes |  | 22 | (42.3) | 18 | (56.2) | 4 | (20.0) | 0.010 |
| **Opioids before simulation, n (%)** | 52 |  |  |  |  |  |  |  |
| no |  | 46 | (88.5) | 26 | (81.2) | 20 | (100.0) |  |
| yes |  | 6 | (11.5) | 6 | (18.8) | 0 | (0.0) | 0.040 |
| **Analgesics during simulation, n (%)** | 52 |  |  |  |  |  |  |  |
| no |  | 49 | (94.2) | 30 | (93.8) | 19 | (95.0) |  |
| yes |  | 3 | (5.8) | 2 | (6.2) | 1 | (5.0) | 0.851 |
| **Analgesics after simulation, n (%)** | 52 |  |  |  |  |  |  |  |
| no |  | 36 | (69.2) | 22 | (68.8) | 14 | (70.0) |  |
| yes |  | 16 | (30.8) | 10 | (31.2) | 6 | (30.0) | 0.924 |

### Supplement figure 1: beach simulation

###
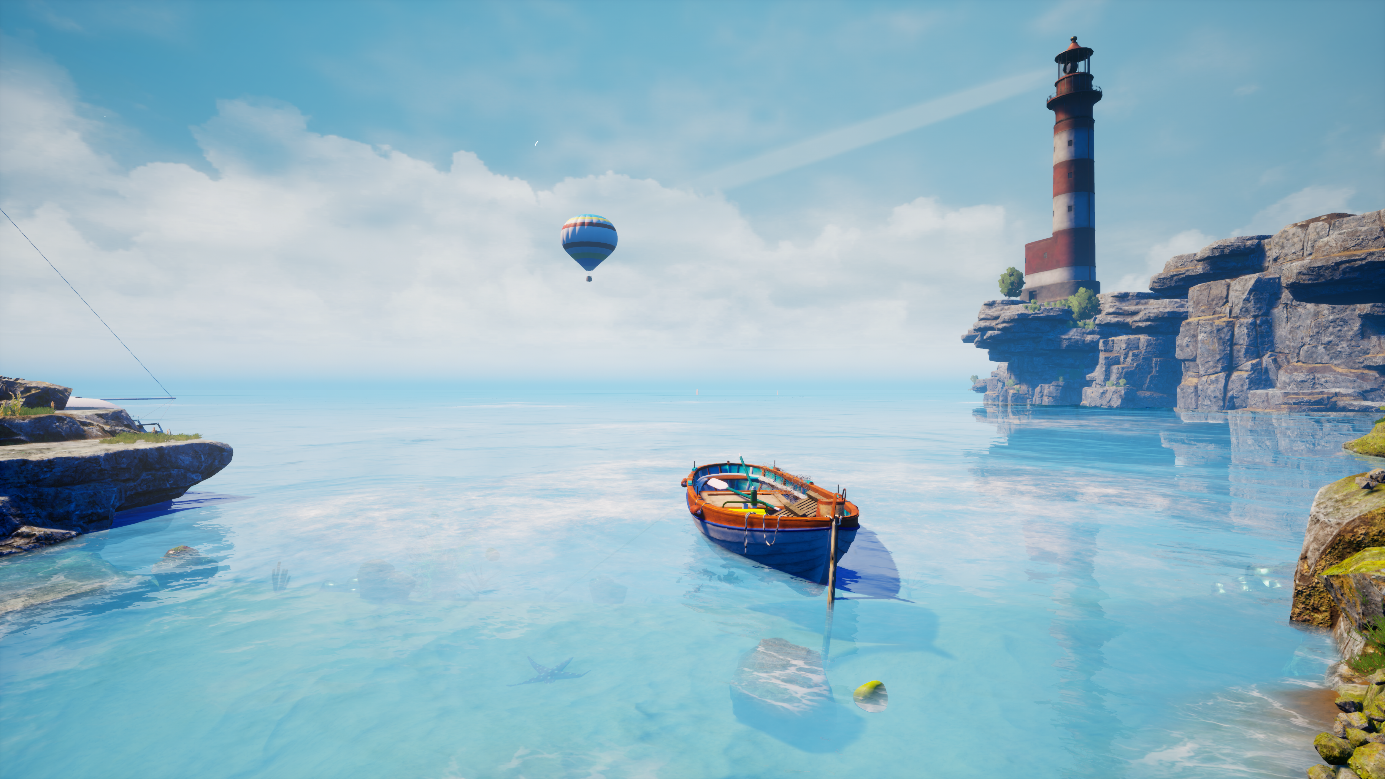


### Supplement figure 2: forest simulation

###
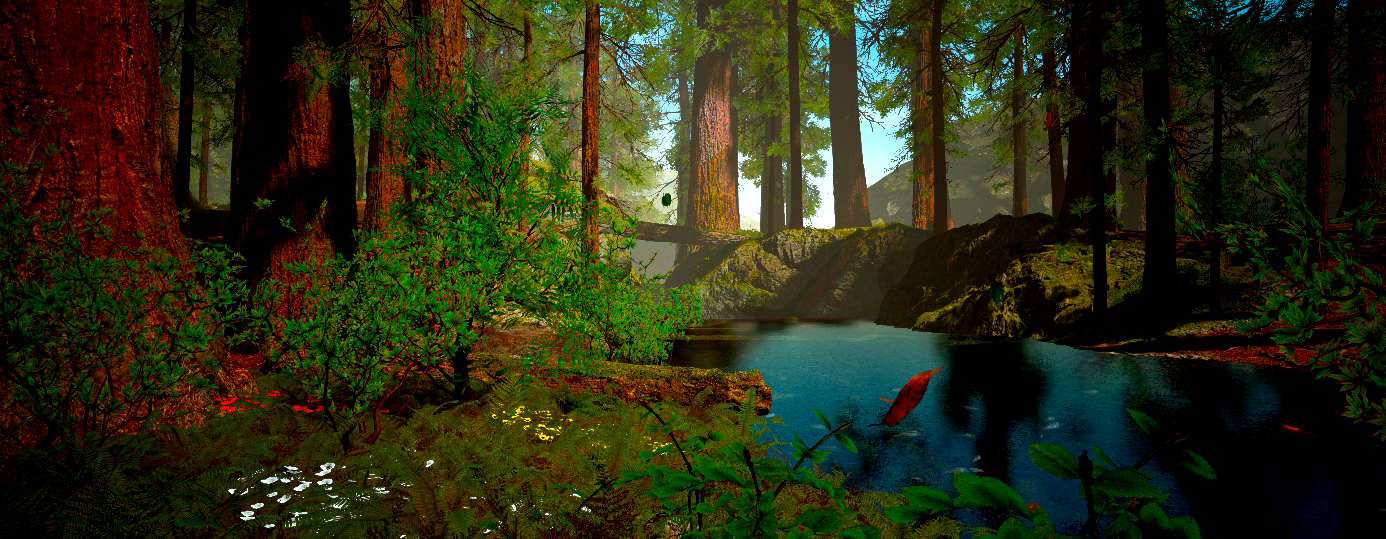

Supplement: Supplementary file 1 — Additional file 1: Supplement table 1. Technical details of the VR simulation. Supplement table 2. Vital parameters before and after the VR simulation. Supplement table 3 Analgesics administered before, during or after the simulation, according to gender. Supplement figure 1. Beach simulation. Supplement figure 2. Forest simulation. [file 12873_2022_671_MOESM1_ESM.docx]
